# Pollution Mitigation in Vermicelli Wastewater: Integrated Fenton and Aerobic Sludge Treatment for Water Quality Improvement

**DOI:** 10.1155/2024/8133617

**Published:** 2024-10-21

**Authors:** Phuoc Bao Niem Nguyen, Van Toan Pham, Hoang Viet Le, Pankaj Kumar, Gowhar Meraj

**Affiliations:** ^1^College of Environment and Natural Resources, Can Tho University, Can Tho 900000, Vietnam; ^2^Institute for Global Environmental Strategies, Hayama 240-0115, Japan; ^3^Department of Ecosystem Studies, Graduate School of Agricultural and Life Sciences, The University of Tokyo, Tokyo 113-0032, Japan

## Abstract

Vermicelli production generates wastewater that is rich in organic and nutrient pollutants, which poses significant environmental challenges. Conventional biological treatments, either alone or in combination with other methods, often fail to achieve high efficiency and operational stability. This study explored the potential of the Fenton process, followed by aerobic activated sludge treatment, to enhance the biodegradability and mineralization of organic substances in vermicelli wastewater. Orientation experiments were performed to examine the effects of operating variables such as pH, reaction time, settling time, and ratio H_2_O_2_/Fe^2+^ on COD removal in order to select the optimal conditions for operating the model in a batch of 20 L, that is, pH = 3, reaction time of 90 min, settling time of 90 min, and ratio of H_2_O_2_/Fe^2+^ used 3 : 1 (4.5 : 1.5 g/L). The removal efficiencies of COD, BOD_5_, TN, TP, and SS reached 75.83%, 67.26%, 28.24%, 26.63%, and 91.9%, respectively. The BOD_5_/COD increased from 0.52 to 0.63, facilitating aerobic activated sludge, which had batch conditions of 15 L with pH of 6.5–8.5, DO ≥3 mg/L, additional nutrients with a dose of 12 mg/L, retention time of 14 h, and settling time of 2 h. As a result, the removal rate of those parameters climbed quite notably, except in SS (95.6%, 96.0%, 84.6%, 84.1%, and 83.6%), and their concentration parameters remained within the allowance levels of the National Technical Regulation in Vietnam before being discharged into the environment. However, the efficiency of treatment in the aerobic activated sludge stage for removing COD and BOD_5_ was not as high as anticipated (83% and 87.33%, respectively) owing to the influence of the high TDS concentration. Thus, additional research is required to address this challenge. The integrated treatment system combining the Fenton process with aerobic activated sludge demonstrated significant potential for the effective reduction of organic and nutrient pollutants in vermicelli wastewater, thereby achieving compliance with regulatory standards. However, the observed limitations in COD and BOD_5_ removal efficiency, likely due to elevated TDS levels, indicate the need for further investigation and optimization to enhance the overall treatment performance.

## 1. Introduction

Wastewater from vermicelli production contains a mixture of many active ingredients. These substances can have harmful effects when discharged directly into the environment. They originate from the process of washing tools, mixing materials, and cleaning production areas, and contain a large amount of starch, sugar, organic acids, and dissolved salts [1, 2]. This wastewater has typical pollution characteristics, such as low pH [3, 4], high chemical oxygen demand (COD) content from 1790 to 3600 (milligram per liter) mg/L [5], high biological oxygen demand (BOD) content from 1070 to 2640 mg/L [5], high suspended solids (SS) content from 414 to 960 mg/L [6], and a relatively high salinity of 3870 ± 226.67 mg/L [7]. If these contaminants cannot be controlled properly, they directly affect the employees of the production facility, surrounding communities, and ecological environment.

The treatment of vermicelli wastewater has been studied, and technological solutions have been proposed previously [6, 8]. In past studies, biological treatment has been widely used to treat wastewater or other types of wastewater with similar characteristics quite effectively [9–13]. However, to achieve a high treatment efficiency, these studies were implemented under various operating conditions, including neutralized pH, dilution with domestic wastewater, retention time from 18 h to 24 h, or combined with other treatment methods, such as flocculation, sedimentation, filtration, up-flow anaerobic sludge blanket (UASB) tank, expanded granular sludge bed (EGSB) anaerobic tank, and internal circulation (IC) anaerobic tank. As a result, operators frequently have to check and complete various steps to ensure the high efficiency of these systems, so the demand to discern novel solutions is essential.

Advanced oxidation processes (APOs) are the most effective solutions for degrading organic pollutants in wastewater, especially recalcitrant macromolecules, by producing highly reactive oxidants, particularly hydroxyl radicals (^∗^OH) [14–17]. One of the most popular APOs for the treatment of organic pollutants is the Fenton reaction. This process produces oxidizing agents through a homogeneous reaction between hydrogen peroxide and ferrous salts in acidic media [18–21]. Compared with other AOPs, treatments based on the Fenton reaction have achieved higher efficiencies [22–24]. This process decomposes a wide range of organic pollutants and generates low-molecular-mass products such as CO_2_ and H_2_O in the case of complete mineralization [25–27]. However, there are limitations to the traditional Fenton reaction, such as the necessity of acidic pH conditions, cost of chemicals, and formation of iron sludge [21, 28, 29]. As such, some methods have been developed to optimize Fenton's treatment and minimize the disadvantages in its operation by using alternative oxidants or catalysts, applying a heterogeneous catalyst, and combining the Fenton process with other methods [30–32].

Taking advantage of the Fenton process to improve the biodegradability index of wastewater, especially those containing bio-recalcitrant organic compounds [33–35], researchers have studied the combination of this process and biotreatment to deal with the restrictions of both methods—consumption of many reagents of the Fenton reaction and time-consuming biological processes [36–38]. Some studies have performed biological treatment and posttreatment using Fenton processes with effective results [39–41]. The main objective of this study was to evaluate the efficiency of the combination of pretreatment by the Fenton process and aerobic activated sludge in the treatment of vermicelli wastewater using a laboratory model.

## 2. Materials and Methods

### 2.1. Data Collection on Vermicelli Production

**Figure 1:**
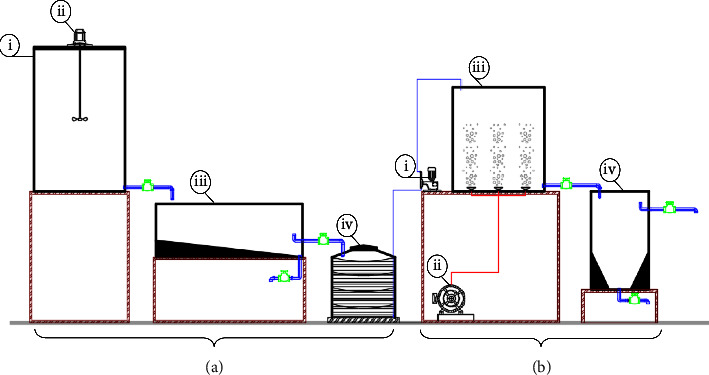
Model diagram of laboratory-scale vermicelli wastewater treatment system. (a) The Fenton stage (i: reaction tank, ii: stirring motor, iii: settling tank, and iv: intermediate tank). (b) The aerobic activated sludge treatment stage (i: pump, ii: blower, iii: aeration tank, and iv: settling tank).

Information on the vermicelli production process, products, and wastewater treatment was collected in 2020. The factory selected for wastewater sampling in this study was a vermicelli production factory in Hau Giang Province, Vietnam. All the information on vermicelli production required in the study was provided by the factory owner. The detailed laboratory-scale vermicelli wastewater treatment system is shown in Figure 1.

### 2.2. Experimental Method

#### 2.2.1. Experiment 1: Treatment Efficiency of Vermicelli Wastewater by the Fenton Method

Orientation experiments were performed in a laboratory reactor, based on a literature review. The most important parameters affecting the efficiency of the Fenton process are the pH, reaction time, settling time, and ratio of H_2_O_2_/FeSO_4_ [25, 42–45]. Subsequently, optimal conditions were selected by evaluating the COD removal efficiency. The experiment was performed in batches, and the volume of wastewater in each treatment batch was 20 L. The oxidation of organic pollutants in the reactor depends on the hydroxyl radical ^∗^OH generated from the reaction between Fe^2+^ and H_2_O_2_. The change in the pH value in the reaction tank was controlled by HCl and NaOH solutions to facilitate the Fenton reaction [46, 47] and stimulate neutralization and flocculation [48, 49]. Then, wastewater was discharged into the settling tank so that sedimentation of Fe (OH)_3_ flocs could occur. The process of mixing the chemicals was performed using a stirrer: the number of revolutions was 50 rpm and the 2-stage stirrer had an angle of 80°relative to the axis. Each experiment was repeated thrice. The average concentrations of the variables were calculated from experimental results. In addition, the model was operated under different conditions to compare the treatment efficiency of the Fenton process and the combination.

#### 2.2.2. Experiment 2: Treatment Efficiency of Vermicelli Wastewater by Combining Two Methods

Based on the results of experiment 1, experiments were performed to evaluate the treatment efficiency of wastewater from vermicelli production using a combination of two processes: the Fenton process and aerobic activated sludge. Raw wastewater was collected in an intermediate tank after pretreatment with the Fenton process. The wastewater was then pumped into the aerobic activated sludge tank in batches with a volume of 15 liter (L). The sludge tank was equipped with a fine-foam aeration system to maintain DO concentration ≥3 mg/L [50], supplemented nutrients by adding nitrogen and phosphorus by chemical fertilizer NPK (N_total_: 20%; P_2_O_5_: 20%; K_2_O: 15%) with a wastewater content of 12 mg/L, maintaining a retention time of 14 h [6], and an activated sludge settling time of 2 h. After settling, the wastewater was collected and analyzed to evaluate the treatment efficiency of the experimental model. Each experiment was repeated thrice, and the average value was calculated from the experimental results.

### 2.3. Chemicals

The chemicals used in the study are summarized in Table 1.

### 2.4. Water Sample Analysis

#### 2.4.1. Sample Collection

Wastewater samples were collected from the collection tank after the preliminary mechanical settling stage of vermicelli production processing to determine the properties of the wastewater from 8 am to 9 am over three consecutive days. Wastewater used for experiments on the Fenton process and the combination process was also collected at the same time and position during the experiment. The pH and total dissolved solids (TDS) were measured in situ. Wastewater samples were stored in plastic cans, cooled in a container with ice, and then transported to the laboratory for analysis. During the experimental period in the laboratory, water sample collection was performed at the input and output of the reactor for each experiment to evaluate the treatment efficiency.

#### 2.4.2. Water Sample Analysis

The physicochemical parameters of water quality, including COD, BOD_5_, total nitrogen (TN), total phosphorus (TP), and suspended solids (SS), were determined by the Standard Methods for the Examination of Water and Wastewater. The pH and total dissolved solids (TDS) parameters were directly measured using the instrument Hq40D-Hatch in situ.

#### 2.4.3. Data Analysis

Descriptive statistical analyses were performed using Microsoft Office Excel 2010. The treatment efficiency of the novel method for treating vermicelli wastewater was evaluated using statistical indicators, including the average and standard deviation. The average values of the physicochemical parameters were selected to operate the model based on the pollutant removal efficiency at the appropriate targets. The quality of the wastewater treated by Fenton's oxidation and aerobic activated sludge was compared with the allowance levels of the national technical regulation on industrial wastewater quality (QCVN 40:2011/BTNMT) promulgated by the Ministry of Natural Resources and Environment, Vietnam [51].

The main objective was to evaluate the treatment efficiency of the Fenton process and the combined treatment process for vermicelli wastewater. Therefore, a deep statistical interpretation of wastewater quality parameters to determine the optimal conditions will be analyzed in future research.

## 3. Results and Discussion

### 3.1. General Information on Vermicelli Production

Vermicelli production is a traditional profession that has existed for a long time in Vietnam and other Asian countries, including Thailand, Cambodia, Malaysia, and China. Together with the development of science and technology, vermicelli production in Vietnam has improved during the production processes, increasing productivity and product quality. According to the information recorded by the authors, the factory had one vermicelli production line with a capacity of 400–600 kilogram per day (kg/day). The production process passes through 10 stages, of which soaking, separating water, and boiling vermicelli directly generate wastewater. Normally, making one batch of vermicelli takes approximately 4 h. The factory could operate the line twice daily. After production is finished, the equipment and production area are cleaned, and these activities also generate wastewater. The total wastewater discharged from all activities of the factory was 5 m^3^/day on average.

### 3.2. Properties of Vermicelli Wastewater

Wastewater from vermicelli production is the amount of liquid discharged during production. Washing and soaking rice are the two main sources of wastewater. This source of wastewater contains starch, trace minerals, vitamins, and suspended solids, which account for approximately 20% to 30% of the total wastewater volume. The water used for washing and cooling the vermicelli after heating was also milky white, but it contained a large amount of starch. Wastewater is also generated from cleaning flour mills, extrusions, and filter cloths. This type of wastewater can contain sand and organic impurities in dissolved or suspended forms, which are mainly carbohydrates such as starch, sugar, and organic acids.

Table 2 shows that the pollution parameters of the vermicelli wastewater, including COD, BOD_5_, SS, and TN, have a high level of concentration. In addition, the TDS parameter is high (>4000 mg/L) because salts and additives are added to create toughness and brightness in the product during the production process of vermicelli. The pH is low because wastewater from rice vermicelli production is acidic due to the long-term soaking of rice, which creates the conditions for the starch hydrolysis process [52]. This highlights the necessity of treating factory wastewater before discharging it into the receiving environment.

### 3.3. Treatment Efficiency of Vermicelli Wastewater by Fenton Method (Experiment 1)

Lab-scale orientation experiments were performed to select the appropriate operation parameters for the Fenton reactor. The treatment efficiency of the Fenton process was evaluated through the COD removal efficiency, as presented in Table 3.

#### 3.3.1. pH

Table 3 shows that COD removal reached its highest efficiency at a pH of 3. This result was quite equivalent to some previous studies that established a pH range of 3.0–3.5 for optimizing COD removal [45, 53, 54]. Moreover, at pH <2.7 and >3.5, the ^∗^OH radical yield decreases significantly [55]. When the pH increases above 3, ferrous ions start to precipitate as Fe(OH)_3_ owing to the reaction with hydroxyl radicals, and the precipitated species are considerably less Fenton-reactive [56, 57], leading to decreased treatment efficiency. Therefore, the pretreatment process in the reactor was performed at a pH of 3.

#### 3.3.2. Reaction Time

There was a slight increase in the treatment efficiency when the reaction time exceeded 90 min. This is because the time required to complete the Fenton reaction depends on numerous factors, especially the dose of the reagents and the contamination level of the wastewater [58, 59]. In addition, to save time and cost, it is not necessary to choose a long reaction time after the degradation efficiency reaches a high level. Therefore, 90 min was selected as the most suitable reaction time to operate the model.

#### 3.3.3. Settling Time

When the settling time was increased from 30 to 45 min after neutralization and flocculation of the Fenton reaction, the treatment efficiency increased from 56.06% to 60.8%, as presented in Table 3. After 45 min of settling, the treatment efficiency slowly increased from 60.8% to 61.74%. In the alkaline solution, Fe^3+^ forms highly insoluble Fe(OH)_3_ to give a flocculent precipitate which facilitates the separation of suspended materials in effluent [60, 61], and this coagulation step of the Fenton process removed both suspended solids in the raw wastewater and partial products of the oxidation reaction, reducing COD concentration [49, 62]. Moreover, after the Fenton reaction, the flow was moved to the settling tank and maintained for approximately 45 min, which allowed enough time for excess H_2_O_2_ to be dispelled; therefore, H_2_O_2_ did not affect the COD concentration. However, the effectiveness of flocculation in reducing COD over time gradually decreased because suspended solids and large colloidal particles settled when they reached a certain size and weight. In wastewater, the only particles that remained were small and lightweight masses because they were too light to settle by gravity or were still not electrically neutral, making them harder to settle [63]. Hence, 45 min were required to operate the pretreatment process in the model.

#### 3.3.4. H_2_O_2_/FeSO_4_

The data in Table 3 indicate that the COD removal efficiency increased notably from 61.01% to 88.08% when the concentration ratio of H_2_O_2_/FeSO_4_ was increased from 1 : 1 to 4 : 1. This trend has been illustrated in previous studies [19, 64] because the increasing H_2_O_2_ concentration generated more ^∗^OH, improving the COD removal rate. After increasing the ratio to 5 : 1, the treatment efficiency slightly increased from 88.08% to 92.18% because, when it reached a certain point, the effect of the oxidant multiple was no longer obvious [65]. It was also found that excess H_2_O_2_ reacted with ^∗^OH radicals, which could be the main reason for the decrease in the number of ^∗^OH radicals [66, 67]. With the H_2_O_2_/Fe^2+^ ratio of 3 : 1 (4.5 : 1.5 g/L), the removal yield COD reached quite a high level of 74.27%, which did not need too much chemicals.

#### 3.3.5. Appropriate Parameters for Pretreatment

Based on previous studies that performed homologous methods with the removal efficiency of COD after the Fenton process ranging from 70% to 78.26% [63, 68] and the results of orientation experiments, the parameters ensured that the influent concentration of COD was suitable for the second stage. They included pH = 3, reaction time of 90 min, H_2_O_2_/Fe^2+^ ratio of 3 : 1 (4.5 : 1.5 g/L), and settling time of 45 min.

**Figure 2:**
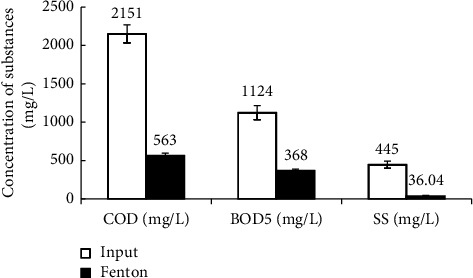
Fenton's removal efficiency of COD, BOD_5_, and SS.

**Figure 3:**
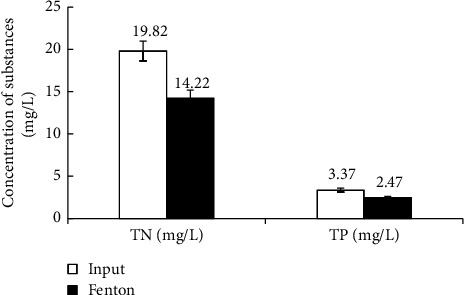
Fenton's removal efficiency of TN and TP.

Figures 2 and 3 show that the removal efficiencies of the physicochemical components COD, BOD_5_, SS, TN, and TP in vermicelli wastewater using the Fenton process reached 75.83%, 67.26%, 91.9%, 28.24%, and 26.63%, respectively. The effective degradation and removal of organic matter is mainly achieved by ^∗^OH oxidation and supplemented by the flocculation and sedimentation of Fe^3+^ complexes. However, these values are still lower than the efficiencies reported in some studies. For instance, COD in the same wastewater is treated using different methods [6, 9, 13] or reversed methods [69, 70]. Moreover, the COD and BOD_5_ concentrations after the Fenton process were higher than the allowance levels of national technical regulations [51]. Notably, the Fenton process was the only pretreatment stage in this study. Therefore, to improve the treatment efficiency of the pollution parameters, further treatment steps should be undertaken.

### 3.4. Treatment Efficiency of Vermicelli Wastewater by Combining Two Methods (Experiment 2)

#### 3.4.1. Aerobic Activated Sludge Biological Treatment

According to Figures 2 and 3, after the Fenton treatment, the concentration of pollutants in wastewater represented by the quality parameters consisting of SS: 36.28 mg/L, BOD_5_: 368.73 mg/L, and the ratio of BOD_5_/COD: 0.63 (>0.5). In addition, to perform coagulation/flocculation for settling with Fe (OH)_3_ flocs, the pH of the wastewater after the Fenton reaction was 7.13. The quality parameters of the wastewater after Fenton's treatment met the conditions for the treatment process using aerobic activated sludge [46]. In addition, the ratio BOD_5_/COD of the wastewater increased from 0.53 to 0.63, because the high molecular weight organic substances were oxidized and short-circuited into low molecular weight biodegradable substances [33, 71], creating favorable conditions for aerobic activated sludge treatment [35, 72]. However, the BOD_5_ : N:P ratio after Fenton treatment was 368 : 14.22 : 2.47, approximately 100 : 3.85 : 0.66, indicating that the wastewater lacked nutrients for biological treatment. Therefore, after Fenton's treatment, the wastewater was enriched with N and P to ensure a BOD_5_ : N:P ratio of 100 : 5 : 1 before starting the activated sludge process [9]. This study did not use halophilic microorganisms that could adapt very well to environments with high TDS concentrations. Therefore, sludge taken from an aerobic treatment tank of a seafood processing wastewater treatment system in a local company was used. This activated sludge was in the log-growth phase and the treatment system of the company was operated for nearly three years, avoiding shock to the activated sludge. Wastewater from the seafood processing factory was selected because its COD and BOD_5_ concentrations are quite similar to those of wastewater after Fenton's treatment [73]. In addition, the study did not focus much on looking for the growth of activated sludge. The experiments included only a few basic nutrients for the rising activated sludge. The activated sludge was grown on an experimental model in which N and P sources were only supplemented by the chemical fertilizer NPK (N_total_: 20%; P_2_O_5_: 20%; K_2_O: 15%). The DO concentration in the aeration tank was maintained at 3-4 mg/L.

According to the mixed liquor suspended solids (MLSS) and mixed liquor volatile suspended solids (MLVSS) content in Table 4, the sludge could adapt and develop into a wastewater treatment system after pretreatment by the Fenton process. The MLVSS content of 72% MLSS was in the suitable range for aerobic activated sludge treatment (MLVSS = 70–80% MLSS) [74, 75]. The sludge continued to grow for 14 d in a tank containing wastewater after treatment by the Fenton process. During the first 7 days, sludge was fed with the low organic loading rate of 0.25 kg/COD.day, and supplemental nutrition with chemical fertilizer NPK at 6 mg/L. In the next 7 days, it was increased progressively up to 0.5 kg/COD.day, and supplementary nutrition up to 12 mg/L. The above processes allow the activated sludge to adapt and increase the amount of biomass in the wastewater after treatment by the Fenton process, allowing the model to be operated and the treatment efficiency to be evaluated [76].

#### 3.4.2. Treatment Efficiency of Combining Fenton Reagent and Aerobic Activated Sludge

Performing all optimal conditions on the model with the combination of two methods and measuring samples after treatment, the removal efficiencies of COD, BOD_5_, TN, TP, and SS were 95%, 96%, 84.6%, 84.1%, and 83.6%, respectively. These results, which are equivalent to those of some studies using different methods of treating vermicelli wastewater or a few types with similar characteristics [9, 11, 12, 77], are also more effective at removing pollutants, especially COD, than other studies [7, 13, 78, 79].

**Figure 4:**
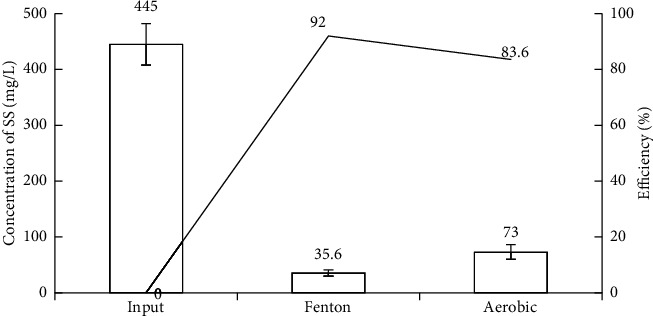
SS removal efficiency of experimental model.

*(1) Removal of SS*. As shown in Figure 4, the SS concentration decreased after treatment with Fenton and increased slightly after treatment with the activated sludge batch. The SS concentration was reduced following Fenton's treatment because neutralization and flocculation occur during the termination of the Fenton reaction [80, 81]. The pH of the solution increased to 7.15, at which Fe^3+^ ions precipitate and Fe (OH)_3_ formed, creating the settling of iron flocs to reduce SS in wastewater. After treatment by the activated sludge, an increase in the SS value was due to the growth of microorganisms that created flocs in wastewater [82, 83]. After settling for 2 h, the SS concentration reached 73 ± 3.20 mg/L and remained within the allowance level of the national technical regulation in Vietnam [51].

The volume of iron sludge created by the Fenton reaction was recorded as high, from 20 to 25% of the total volume batch treatment (20 L), but there were no detailed parameters compared to the literature [84]. Therefore, further research is required to more precisely calculate the amount of sludge necessary to choose the most suitable methods for treatment, such as dried yard or press machine, and will require further research on other processes to replace the conventional Fenton process by the electro-Fenton process, photo-Fenton process, fluidized-bed Fenton process, or heterocatalyst types in the Fenton processes to reduce the volume of sludge [85–87].

**Figure 5:**
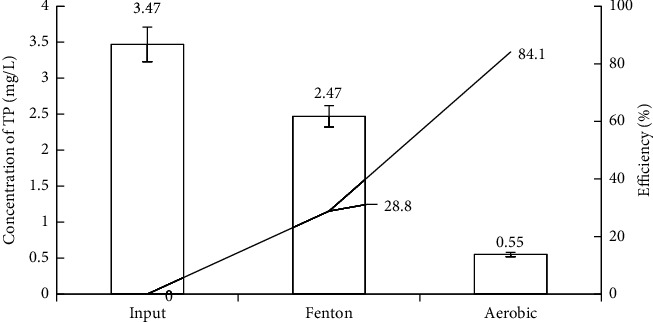
TP removal efficiency of experimental model.

**Figure 6:**
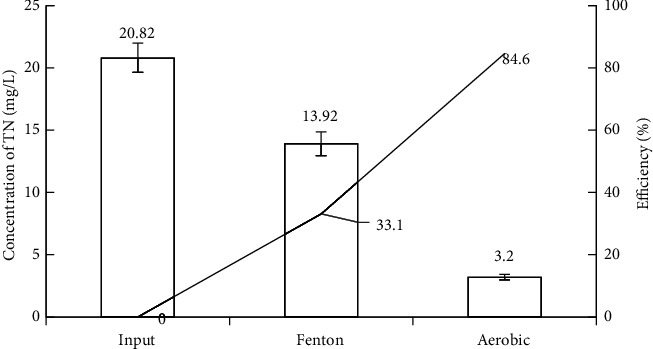
TN removal efficiency of experimental model.

*(2) Removal of TN and TP*. Because of the specific characteristics of vermicelli wastewater with lower concentrations of TN and TP, the treatment of these two parameters was rather convenient. According to the results after Fenton treatment in Figures 5 and 6, the TN and TP concentrations decreased mainly because of the neutralization process and flocculation after the Fenton reaction ended.

Moreover, some Fe^3+^ ions reacted with P-PO_4_^3-^ ions to precipitate FePO_4_; thus, the TP concentration decreased slightly [88]. When conducting treatment with activated sludge, appropriate quantities of additional nutrients from NPK fertilizers were added to ensure the growth of microorganisms [89] and avoid odd concentrations causing eutrophication in the receiving water. As a result, the output wastewater had low concentrations of TN (3.2 ± 0.22 mg/L) and TP (0.55 ± 0.03 mg/L) corresponding to the treatment efficiency of 84.6% and 84.1%, respectively. The concentrations of TN and TP also remained at the allowance level of QCVN 40:2011/BTNMT [51] and the wastewater discharge standards of some countries such as the United States of America, Canada, and Germany [90].

**Figure 7:**
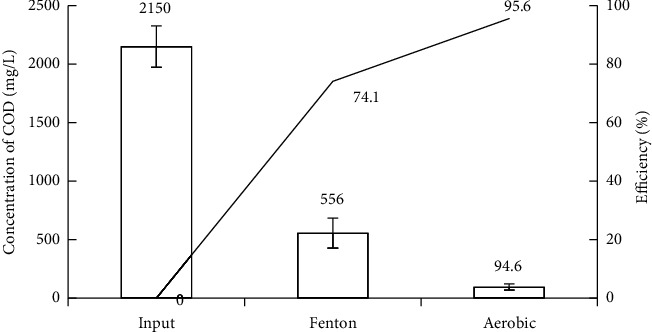
COD removal efficiency of experimental model.

**Figure 8:**
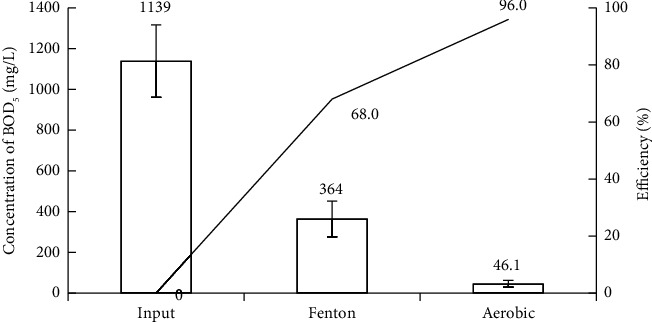
BOD_5_ removal efficiency of experimental model.

*(3) Removal of COD and BOD_5_*. According to Figure 7, the concentration of COD in the wastewater treated by the experimental model decreased significantly, resulting in a high efficiency of 95.6%. Moreover, Figure 8 shows an overwhelming decrease in the concentration of BOD_5_, and its efficiency increased notably to 96%. The effective removal of COD and BOD_5_ was achieved by the Fenton reaction, which consists of chain processes [15]. Equation (1) is usually considered for the core [86]:

(1) $$\begin{array}{c} {\text{Fe}}^{2+}+H_{2}O_{2}⟶{\text{Fe}}^{3+}+\cdot \text{OH}+{\text{OH}}^{-} \end{array}$$

This step also creates mainly ^∗^OH [91] that can oxidize organics (RH), particularly recalcitrant organic compounds, by eliminating protons, resulting in potentially reactive organic radicals (R•) that can be oxidized further [92] and even completely mineralize them into CO_2_ and H_2_O [85]:

(2) $$\begin{array}{c} \text{RH}+{\text{OH}}^{•}⟶H_{2}O+{\text{CO}}_{2}+R^{•}⟶\text{further oxidation} \end{array}$$

Based on the reaction as equation (2), the COD and BOD_5_ concentrations in wastewater were quite significantly reduced by 1594 mg/L and 775 mg/L, respectively, and the ratio BOD_5_/COD of the wastewater was increased from 0.53 to 0.65 by improving the biodegradability of wastewater [93, 94]. The products were obtained after Fenton treatment using aerobic microorganisms of the activated sludge in the next stage:

(3) $$\begin{array}{c} R^{•}+O_{2}+\text{aerobic microorganisms}⟶{\text{CO}}_{2}+{\text{NH}}_{4}^{+}+\text{other products}+Q \end{array}$$

(4) $$\begin{array}{c} R^{•}+O_{2}+\text{aerobic microorganisms}+Q⟶C_{5}H_{7}O_{2}N\;\left(\text{new bacteria cells}\right) \end{array}$$

These activities of aerobic microorganisms as equations (3) and (4) resulted in the reduction of organic pollutants [95], leading to decreasing the concentration of COD and BOD_5_ by 461.4 mg/L and 317.9 mg/L, respectively, via the settling of activated sludge flocs in the settling tank. Their concentrations also remained at the allowance levels of QCVN 40:2011/BTNMT [51].

*(4) Influence of TDS to Aerobic Activated Sludge Stage*. The removal efficiencies of COD and BOD_5_ from the aerobic activated sludge stage were not as high as expected, at 83% and 87%, respectively, and these results were also lower than those of some studies using biotreatment for vermicelli wastewater [6, 9, 11]. It is because the vermicelli production wastewater has high TDS concentrations of 4532.67 ± 216.04 mg/L due to using dissolved ions such as Na^+^, Cl^−^, Mg^2+^, and K^+^ in the production process. On the other hand, during the Fenton process, chemicals were added to adjust and participate in the reaction process, such as HCl, FeSO_4_, and NaOH, and could therefore mineralize organic pollutants into inorganic salts [86]. These substances contributed to increasing the content of dissolved substances in the wastewater. Although some of the chemicals were removed during the settling process, they still made the TDS concentration after the treatment increased to approximately 5000 mg/L. A high TDS concentration could cause osmotic pressure or inhibit the growth of aerobic activated sludge, which reduces the COD and BOD_5_ removal efficiency of the aerobic activated sludge method [96–98]. However, the retention time of the aerobic activated sludge stage was 14 h; therefore, the total time-consuming treatment of the whole process was approximately 16 h. It is shorter than that in studies with similar efficiency when using traditional methods for vermicelli wastewater, such as anaerobic-aerobic digestion sequencing (32 h) and aerobic multisteps (20 h) [6], settling–aerobic (39 h) [9], and anaerobic trickling filter with rotating biological contactor (32 h) [11]. Moreover, to improve the efficiency of pollutant removal from vermicelli wastewater and reduce the chemical cost, further research could present a few solutions. In the pretreatment stage, advanced oxidation processes such as photo-Fenton, photo-Fenton-like, electro-Fenton, and H_2_O_2_/catalyst processes could supersede the conventional Fenton process [85, 99, 100]. For the aerobic activated sludge stage, halophilic microorganisms capable of withstanding high TDS concentrations are necessary [97, 101, 102].

#### 3.4.3. Comparison between Fenton Process Alone and the Combination of Two Methods

The effectiveness of the combined treatment method and the Fenton process for removing pollutants from vermicelli wastewater was compared. The results of the comparison of the pollution parameters are presented in Table 5.

Table 5 indicates that the concentration of pollutants in wastewater after treated by the combination was almost lower than that by the Fenton process, except SS due to the effect of the biotreatment stage. Moreover, the COD and BOD_5_ in wastewater after the Fenton process exceeded the allowance level of QCVN 40:2011/BTNMT [51]. The Fenton process consumed chemicals more than the combination. If the experiment continued to increase the ratio H_2_O_2_/Fe^2+^, the quality of water would be better, but it had to deal with the high cost of the chemicals.

## 4. Conclusion

This study demonstrated the effectiveness of a combined Fenton process and aerobic activated sludge treatment for the remediation of vermicelli production wastewater. The combined method achieved significant reductions in key pollutants, including COD and BOD_5_, thereby meeting regulatory standards for wastewater discharge. The notable advantages of this method include its shorter treatment time and high removal efficiency, particularly for challenging organic loads. The applicability of this combined treatment approach extends beyond vermicelli wastewater to other industries with similar effluent characteristics, such as those involving rice noodles, sweet potato starch, the bakery industry, Thai fermented rice noodles, and corn starch. This versatility underscores the potential for broader adoption in various food processing sectors. Despite these promising results, several limitations were identified. The presence of high total dissolved solids (TDS) posed challenges for the biological treatment phase, potentially inhibiting microbial activity and reducing overall treatment efficacy. Additionally, the generation of iron sludge during the Fenton process requires careful management and disposal, adding to the operational complexity and costs. Future research should focus on optimizing the treatment process by exploring advanced oxidation techniques, such as photo-Fenton and electro-Fenton processes, to enhance the degradation of recalcitrant pollutants while minimizing chemical consumption. Additionally, the integration of halophilic microorganisms in the aerobic treatment stage could mitigate the adverse effects of high TDS levels. Long-term studies and pilot-scale implementations will be critical to assess the economic feasibility, environmental impact, and practical scalability of this combined treatment approach in industrial settings.

## Figures and Tables

**Table 1:** Chemicals used in the study.

Reagents	Manufactures	Purity (%)
*Used for operating the treatment system*		
FeSO_4_.7H_2_O	Sunkan Chemicals (China)	98
H_2_O_2_	Taekwang Industrial (Korea)	50
NaOH	Gansu Nabowang Chemical (China)	99
HCl	Viet Tri Chemical (Vietnam)	35

*Used for analyzing physicochemical variables*		
All chemicals used for analysis	Merck (Germany)	≥99

**Table 2:** The characteristics of vermicelli wastewater.

Parameters	Unit	Values
pH	—	4.12
COD	mg/L	2148.68 ± 101.6
BOD_5_	mg/L	1159.80 ± 54.33
SS	mg/L	430.01 ± 28.01
TDS	mg/L	4475 ± 226.84
TN	mg/L	19.82 ± 1.17
TP	mg/L	3.37 ± 0.24

**Table 3:** Removal efficiency of COD corresponding to operation parameters.

pH	2	2.5	3	3.5	4	4.5
COD removal efficiency (%)	46.31	49.61	54.62	44.01	41.82	36.58

Reaction time (min)	30	45	60	90	120	
COD removal efficiency (%)	49.83	52.84	55.01	60.7	62.24	

Settling time (min)	30	45	60	90	120	
COD removal efficiency (%)	56.06	60.08	61.19	61.37	61.74	

Ratio H_2_O_2_/Fe^2+^	1 : 1	2 : 1	3 : 1	4 : 1	5 : 1	
COD removal efficiency (%)	61.01	64.35	74.27	88.08	92.18	

*Note.* (i) After each orientation experiment, an optimal parameter was selected applying for the next experiments. (ii) Ratio H_2_O_2_/Fe^2+^ 1 : 1 = 1.5 : 1.5 (g/L).

**Table 4:** The MLSS and MLVSS content of activated sludge by batch.

	Unit	12 h	24 h
MLSS	mg/L	2257.5 ± 6.2	2378.2 ± 13.67
MLVSS	mg/L	1668.9 ± 19.53	1723.9 ± 5.98

**Table 5:** The concentration of pollutants in wastewater after treatment of two experiments.

Parameters	Unit	Fenton process	Combination
COD	mg/L	154.52 ± 7.30	94.65 ± 4.72
BOD_5_	mg/L	83.49 ± 4.53	46.14 ± 2.02
TN	mg/L	13.61 ± 0.98	3.20 ± 0.22
TP	mg/L	2.61 ± 0.15	0.55 ± 0.03
SS	mg/L	44.01 ± 3.23	73.00 ± 3.20

## Data Availability

Data are available on request from the corresponding author.
